# Management of mandibular angle fractures through single and two mini-plate fixation systems: Retrospective study of 112 cases

**DOI:** 10.1016/j.ijscr.2021.105690

**Published:** 2021-02-23

**Authors:** Ouassime Kerdoud, Rachid Aloua, Amine kaouani, Faiçal Slimani

**Affiliations:** a Faculty of Medicine and Pharmacy, Hassan II University of Casablanca, B.P 5696, Casablanca, Morocco; b Oral and Maxillofacial Surgery Department, CHU Ibn Rochd, B.P 2698, Casablanca, Morocco

**Keywords:** Osteosynthesis, Complications, Standard miniplate, Mandibular angle fracture, Rigid fixation, Surgical treatment

## Abstract

**Introduction:**

The aim of this paper is to compare the clinical results between the different methods of fixation of standard mini-plates for the management of mandibular angle fractures.

**Study design:**

This was a retrospective analysis study of 196 patients with mandibular angle fractures divided into 3 groups at our hospital 20 august 1953 specialist hospital, which is a referral center between January 2015 and January 2020. The patients were assessed for malocclusion, infection, wound dehiscence, neuro-sensory deficit, mouth opening, stability, operating time, blood loss, and hardware failure. Group (I) comprised 72 men and 40 women with a mean age of 43 years. Group (II) comprised 36 men and 12 women with a mean age of 47 years. Group (II) comprised 29 men and 7 women with a mean age of 33 years. The assessment of surgical outcomes after the last follow-up visit clearly showed a lack of stability in patients group II compared to the other groups. The operating time was reduced in group I compared to Group II/ III. (P = 0.03) The wound dehiscence occurred mostly in Group III unless it was statistically no significant. The infection events occurred in 36 patients of the sample, which was not statistically significant (Table 4).

**Discussion:**

Fractures of the mandibular angle represent 23–42% of all mandibular fractures, in our context road accidents followed by aggression are the most frequent mechanisms found. A various types of treatment approaches for the treatment of angular fractures have been described. Our analysis revealed that surgical time and complication rate has been reduced when using the Group I technique compared to the other groups.

**Conclusion:**

The use of the monocortical external oblique miniplate provides the best results. Successful treatment of mandibular fractures depends on stability in the ideal anatomical position since abnormal mobility at the fracture site will lead to non-union, malocclusion, and infection.

## Highlights

• The management of mandibular angle fractures is often challenging and has a high complication rate.
• The treatment of mandibular angle fractures aims to achieve a good reduction, stable fixation, and early recovery of masticatory function.
• The contemporary practice uses a variety of surgical techniques for the fixation of angular fractures.
• The single miniplate has recently become the technique of choice.

## 1 Introduction

The management of mandibular angle fractures is often challenging and has a high complication rate. The most appropriate treatment of angle fractures remains controversial [1,2].

Biomechanical analysis has revealed that the best site for plating is the flat, vestibular bone part located in the third molar region. The easiness of access and the extreme strength of the cortex make this site the preferred site for angle osteosynthesis. However, lower osteosynthesis, on the external surface of the mucosa, is sufficiently strong to support the stress generated by masticatory forces in the angular region [3,4].

The mandibular angle area is submitted to biomechanical forces (due to muscle insertion) and the presence of the third molar [5], therefore the treatment of mandibular angle fractures requires an in-depth understanding of the anatomy of the region. The treatment of mandibular angle fractures aims to achieve a good reduction, stable fixation, and early recovery of masticatory function [6].

The contemporary practice uses a variety of surgical techniques for the fixation of angular fractures. The single noncompression monocortical miniplate fixation of the angular fractures has recently become the technique of choice [7].

## 2 Patients and methods

This was a retrospective analysis study of 196 patients with mandibular angle fractures divided into 3 groups at our hospital 20 august 1953 specialist hospital, which is a referral center between January 2015 and January 2020. All patients underwent an open reduction under general anesthesia. The surgeries were performed by a team of residents under the supervision of the chief professor of the maxillofacial surgery department.

Inclusion criteria were patients diagnosed with angular fractures of the mandible and surgically treated with open reduction.

Patients with any associated mandibular fractures, patients not fit to undergo procedures under general anesthesia, treated by orthopedic approach were excluded from this study.

This study's data were collected using the files' analysis focused on the epidemiological, clinical, radiological explorations, therapeutic aspects. The patients were assessed for malocclusion, infection, wound dehiscence, mouth opening, stability, operating time, blood loss, and hardware failure.

Data management and analysis were performed using IBM SPSS Statistics for Windows, version 25.0.0 (IBM Corporation, Armonk, NY). Categorical data were summarized as frequencies, and cross-tabulations and x2 tests for significance made comparisons across allocated groups. Continuous variables were summarized as the mean and range, and comparisons between groups were made using the ANOVA test. All significance tests used a two-sided P-value of 0.05.

This case series has been reported in line with the PROCESS criteria [8].

## 3 Surgical technique

After placement of arch bars, the surgical incision was performed. A reduction of fracture is done, and the jaws were placed into postoperative maxilla-mandibular fixation if it’s necessary (MMF).

Group I: A single 4-hole non-compression miniplate (2 mm) was fixed in the external oblique line at the superior border of the mandible (Picture 1).

**Picture 1:**
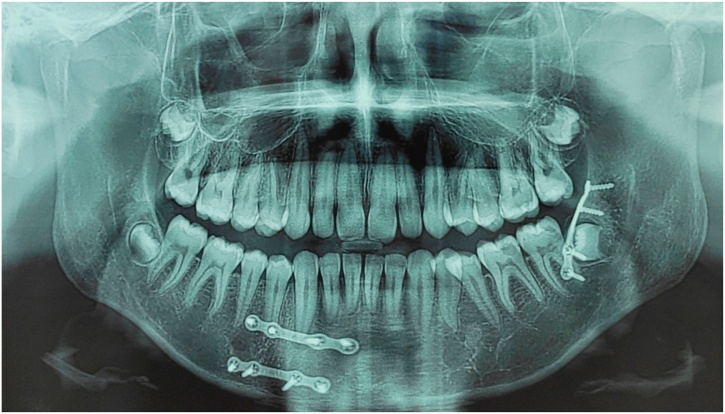
A single 4-hole non-compression miniplate (2 mm) was fixed in the external oblique line at the superior border of the left mandible.

Group II: A single 4-hole non-compression miniplate (2 mm) was fixed at the ventral border of the mandible (Picture 2).

**Picture 2:**
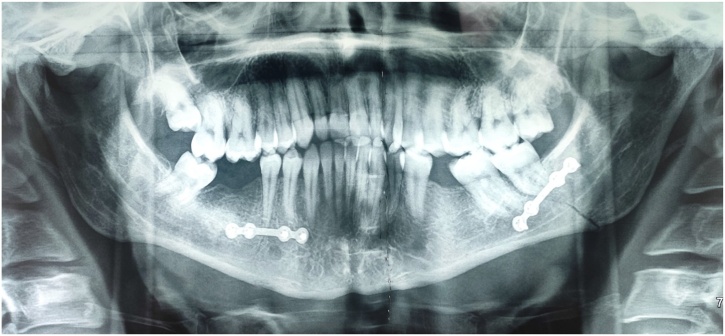
A single 4-hole non-compression miniplate (2 mm) was fixed at the ventral border of the left mandible.

Group III: two 4-hole non-compression mini plates in which 1 plate was fixed like that in group I and the other plate was fixed to the lateral aspect of the angle of the mandible (Picture 3).

**Picture 3:**
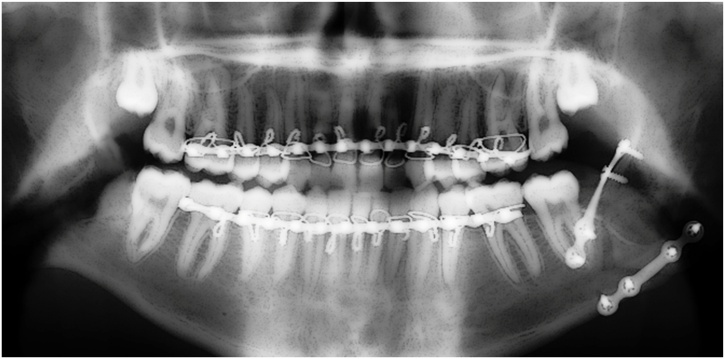
Two 4-hole non-compression mini plates in which 1 plate was fixed like that in group I and the other plate was fixed to the lateral aspect of the angle of the mandible.

After the plates were placed, MMF was released and occlusion was checked. The intraoral incision was closed with resorbable sutures. Patients were reviewed at 1, 2, 4, and 6 weeks after surgery. The arch bars were removed after the fourth postsurgical week. The patients received amoxicillin/clavulanic acid 1 g twice daily and antalgics for 8 days.

To examine the neuro-sensory deficit, the ability to feel touch or pain in the lower lips was tested. Our patients' eyes were closed. Using a piece of cotton, we applied a light touch to one or both sides of the third trigeminal division and asked the patient to show or tell if the sensation is the same on both sides.

## 4 Results

The characteristics of the patients divided into groups, as well as the clinical considerations are presented in Table 1.

**Table 1:** Characteristic of Population.

	Groups		
	G(1)	G(2)	G(3)
Mean age (yr.)	43±19	47±17	33±12
Gender			
Female	40	12	7
Male	72	36	29
Oral hygiene			
Poor	20	11	16
Average	68	18	10
good	24	29	10
TOTAL	112	48	36

We included in Group (I), 112 patients who were treated with a single non-compression mini-plate fixed in the oblique line at the superior border through an intraoral approach. In Group (II), 48 patients were treated with a mini-plate fixed to the lateral aspect of the angle of the mandible with the intraoral approach. 36 patients were included in the group (III) which two mini-plates in which one miniplate was fixed like that in the group (I) and the other was fixed in the ventral aspect of the angular area of the mandible.

Group (I) comprised 72 men and 40 women with a mean age of 43 years.

Group (II) comprised 36 men and 12 women with a mean age of 47 years.

Group (II) comprised 29 men and 7 women with a mean age of 33 years (Table 2).

**Table 2:** Comparison among the groups depending on occlusion, mouth opening, infection, stability at the 7th post-op days.

	Occlusion		Mouth opening		Stability	
	Satisfactory (%)	Deranged (%)	Adequate (%)	Inadequate (%)	Stable (%)	Unstable (%)
Group I (n = 112)	91 (81.25)	21 (18.75)	103 (91.96)	9 (8.04)	95 (84.82)	17 (15.18)
Group II (n = 48)	19 (39.58)	29 (60.42)	31 (64.58)	17 (35.42)	10 (20.83)	38 (79.17)
Group III (n = 36)	24 (66.67)	12 (33.33)	22 (61.11)	14 (38.89)	32 (88.89)	4 (11.11)
P value	0.021		0.843		0.07	

The assessment of surgical outcomes after the last follow-up visit clearly showed a lack of stability in patients group II compared to the other groups. The operating time was reduced in group I compared to Group II/ III. (P = 0.03) (Table 3).

**Table 3:** Comparison among the groups depending on operating time, blood loss, hospital stay.

	Operating time (mean minutes)	Blood loss (ml)	Hospital stay (mean)	Postoperative MMF (n)
Group (I)	33 ± 12	50	2.33 ± 1.27	45
Group (II)	43 ± 11	55	2.44 ± 1.02	15
Group (III)	83 ± 21	91	3,78 ± 2.45	24
P value	0.03	0.042	0.06	0.89

The wound dehiscence occurred mostly in Group III unless it was statistically no significant. The infection events occurred in 36 patients of the sample, which was not statistically significant (Table 4).

**Table 4:** Comparison among the groups depending on occlusion, mouth opening, infection, stability.

	Wound dehiscence (%)		Hardware failure (%)		Non-union (%)		Neuro-sensory deficit (%)		Infection (%)	
	yes	no	yes	no	yes	no	yes	no	yes	no
Group I (n = 112)	17 (15.18)	95 (84.82)	3 (2.68)	109 (97.32)	8 (7.14)	104 (92.86)	34 (30.36)	78 (69.64)	13 (11.6)	99 (88.4)
Group II (n = 48)	7 (14.58)	41(85.42)	9 (18.75)	39 (81.25)	7 (14.58)	41 (85.42)	16 (33.33)	32 (66.67)	17 (35.42)	31 (64.58)
Group III (n = 36)	22 (61.11)	14 (38.89)	7 (19.44)	29 (80.56)	7 (19.44)	29 (80.56)	29 (80.56)	7 (19.44)	6 (16.67)	30 (83.33)
P value	0.08		0.32		0.076		0.057		0.066	

Routine follow-up 1, 3, 6, and 12 months in our specialized consultation; any clinical signs that appeared were mentioned on the patient's discharge form. Mild edema and paresthesia were common during the first week postoperatively in our study; no vascular damage was noted. Nineteen patients from all groups required plate removal given the evolution towards cervicofacial cellulitis.

## 5 Discussion

Fractures of the mandibular angle represent 23–42% of all mandibular fractures, in our context road accidents followed by aggression are the most frequent mechanisms found. Unerupted and impacted wisdom teeth create an area of weakness. A various types of treatment approaches for the treatment of angular fractures have been described [9].

Successful treatment of mandibular fractures depends on stability in the ideal anatomical position since abnormal mobility at the fracture site will lead to non-union, malocclusion, and infection [10].

The first classification of nerve injury was established by Seddon in 1947, who described nerve damage in three grades of injury [11]. In 1951, Sunderland enlarged the histologically based classification to include five degrees of lesions, which corresponded to Seddon's three-level classification overall, with a more accurate prognosis of the outcome of axonotrimary lesions [12] (Table 5).

**Table 5:** Nerve injury classification in increasing severity.

Sunderland [12]	Seddon [11]	Features
Type 1	Neuropraxia	Damage to local myelin only
Type 2	Axonotmesis	Division of intraneural axons only
Type 3	Axonotmesis	Division of axons and endoneurium
Type 4	Axonotmesis	Division of axons, endo- and perineurium
Type 5	Neurotmesis	Complete division of all elements including epineurium
Type 6	Mixed	Combination of types 2–4

The aim of this paper was to check by retrospective study, whether there is a significant difference in clinical outcome between the different fixation methods in the management of angular fractures of the mandible.

Our analysis revealed that surgical time and complication rate has been reduced when using the Group I technique compared to the other groups, but recently, based on advanced design and modeling of 3D mini-plates using the finite element method combined with CAD/CAM technology that could further reduce surgical time, since the surgeon would not have to model the plates intra-operatively [[13], [14], [15]].

The difference between the techniques regarding the incidence of wound dehiscence (15.17% in Group I) may be related to the proximity of the mini-plate to the incision, when placed on the external oblique line. However, the differences between the groups were not statistically significant (P = 0.08).

No statistically significant difference in the incidence of paresthesia was observed between the three techniques (P = 0.057). In the course of the operation, aggressive manipulation due to the displacement of the fracture may account for additional nerve damage. Therefore, it is important to seek paresthesia before surgery because if it’s not checked before the operation, it may appear as an iatrogenic complication of the surgery.

The follow-up period in the study was 1–12 months. Several complications may not appear immediately, such as plaque exposure or infection, which may occur months or years after successful healing [16].

## 6 Conclusion

In summary, the results of this study identified lower complication rates with the use of oblique line mini-plate fixation compared to other methods of standard miniplate fixation in the management of angular fractures of the mandible.

## Declaration of Competing Interest

The authors report no declarations of interest.

## Funding

The authors declared that this study has received no financial support.

## Ethical approval

Written informed consent was obtained from the patient for publication of this case report and accompanying images. A copy of the written consent is available for review by the Editor-in-Chief of this journal on request.

## Consent

Written informed consent was obtained from the patient for publication of this case report and accompanying images. A copy of the written consent is available for review by the Editor-in-Chief of this journal on request.

## Author contribution

Ouassime kerdoud: Corresponding author writing the paper.

Rachid Aloua: writing the paper.

Amine kaouani: writing the paper.

Faiçal Slimani: Correction of the paper.

## Registration of research studies

researchregistry6471.

## Guarantor

Ouassime Kerdoud.

## Provenance and peer review

Not commissioned, externally peer-reviewed.
